# Spike proteins of coronaviruses activate mast cells for degranulation via stimulating Src/PI3K/AKT/Ca^2+^ intracellular signaling cascade

**DOI:** 10.1128/jvi.00078-25

**Published:** 2025-04-30

**Authors:** Shuang Zhang, Chu-Lan Xu, Jingjing Wang, Xiaoli Xiong, Jian-Hua Wang

**Affiliations:** 1 Guangzhou Institutes of Biomedicine and Health, Chinese Academy of Sciences 74627, Guangzhou, China; 2 State Key Laboratory of Antiviral Drugs, Pingyuan Laboratory, Henan Normal University 66519, Xinxiang, Henan, China; The Ohio State University, Columbus, Ohio, USA

**Keywords:** mast cell, degranulation, coronavirus

## Abstract

Mast cells (MCs) are strategically located at the interface between host and environment. The non-allergic functions of MCs in immunosurveillance against pathogens have been recently underscored. However, the activation of MCs by pathogens may beneficially or detrimentally regulate immune inflammation to combat or promote pathogen invasion. We and others have conclusively demonstrated that MCs serve as a crucial mediator in the induction of hyperinflammation initiated by severe acute respiratory syndrome coronavirus 2 (SARS-CoV-2), leading to substantial tissue damage across multiple organs in murine and nonhuman primate models. Whereas the precise mechanism underlying virus-induced MC activation and degranulation remains largely elusive, our previous findings have indicated that the binding of the Spike proteins to cellular receptors is sufficient to elicit MC activation for rapid degranulation. This study aims to corroborate the ubiquity of coronavirus-induced MC degranulation and elucidate the intracellular signaling pathways that mediate the activation of MCs upon Spike protein binding to the cellular receptors. Our transcriptome analysis revealed MC activation upon the stimulations with a range of Spike/RBD proteins and viral particles of coronavirus. Notably, the interaction between these Spike/RBD proteins and cellular receptors triggered the activation of src kinase, a member of Src Family Kinases (SFKs). This activation, in turn, stimulated the PI3K/AKT signaling pathway, resulting in an accumulation of intracellular calcium ions. These calcium ions subsequently facilitated microtubule-dependent granule transport, ultimately promoting MC degranulation. In summary, this study elucidates the mechanism underlying virus-triggered activation of MCs and has the potential to aid in the development of MC-targeted antiviral therapeutic strategies.

**IMPORTANCE:**

The activation and degranulation of mast cells (MCs), triggered by a variety of viruses, are intricately linked to viral pathogenesis. However, the precise mechanism underlying virus-induced MC degranulation remains largely unknown. In this study, we demonstrate the ubiquity of coronavirus-induced MC degranulation and investigate the intracellular signaling pathways that mediate this process. We reveal that the binding of Spike proteins and cellular receptors is sufficient to elicit MC activation for rapid degranulation. This binding triggers the activation of src kinase and the downstream PI3K/AKT cellular signaling pathway, resulting in an accumulation of intracellular calcium ions. These calcium ions subsequently facilitate microtubule-dependent granule transport, ultimately promoting MC degranulation. This study elucidates the mechanism underlying virus-triggered activation of MCs and has the potential to aid in the development of MC-targeted antiviral therapeutic strategies.

## INTRODUCTION

MCs are strategically located at the interface between host and environment. Their well-documented function is acting as the primary effector cells in type I hypersensitivity reactions (1). However, recent research has increasingly highlighted their non-allergic roles in immune surveillance against a broad spectrum of pathogens, including bacteria, parasites, and viruses (2–6). MCs function as sentinels to recognize invaded pathogens and regulate the innate and adaptive immune responses (1, 6–8). MCs express a range of receptors to recognize and bind to a variety of ligands, including microbial components. Upon activation, MCs undergo a series of functional changes, including the secretion of cytokines, chemokines, and other inflammatory mediators, which drive the recruitment and activation of other immune cells (3–6, 9).

The interactions between MCs and a variety of viruses, including respiratory syncytial virus, rhinovirus, reovirus, dengue virus, human immunodeficiency virus (HIV), influenza viruses, and hepatitis viruses, have been reported (4, 6, 10, 11). The activated MCs regulate antiviral immunity through the release of mediators or via direct interaction with other immune cells (6, 12, 13). However, it is noteworthy that inappropriate inflammatory responses induced by MCs may result in tissue hyperinflammation, vascular leakage, and the impairment of host immunity, ultimately facilitating viral invasion (6, 12, 13). In the context of coronavirus infection, we and others have recently elucidated the pivotal role of MCs as a crucial mediator in the hyperinflammatory response initiated by SARS-CoV-2. Specifically, the infection of SARS-CoV-2 triggers substantial accumulation and rapid degranulation of MCs, leading to inflammation and tissue damage in the lungs, brain, trachea, and bronchi of murine and nonhuman primate models (14–17). Furthermore, the systemic inflammation induced by MCs has been implicated in the pathogenesis of long-COVID (18). These observations underscore the significance of MCs in the pathogenesis of SARS-CoV-2-induced inflammation. Additionally, we have observed that other coronaviruses are also capable of inducing MC activation (14). However, the underlying mechanisms responsible for pathogen-induced MC activation and degranulation remain largely elusive.

MCs express a diversity of membrane receptors, including FcεRI, Toll-like receptors (TLRs), complement receptors (CR1-5), and IgG receptors (FcγRI and FcγRII), etc., which serve as the binding sites for pathogens or their derivatives to elicit MC activation (1, 8). In the context of allergic reactions, IgE/allergen-mediated activation of FcεRI triggers intricate cytoplasmic signaling cascades, ultimately culminating in vesicular transport and the *de novo* synthesis of mediators within MCs (1, 8, 19). Our previous findings have indicated that the binding of the Spike proteins with cellular receptors is sufficient to elicit MC activation for rapid degranulation (14). This study aims to corroborate the ubiquity of coronavirus-induced MC degranulation and elucidate the intracellular signaling pathways that mediate the activation of MCs upon Spike protein binding to the cellular receptors.

## RESULTS

### The binding of Spike/RBD proteins of coronaviruses to receptors triggers MC degranulation

**Fig 1:**
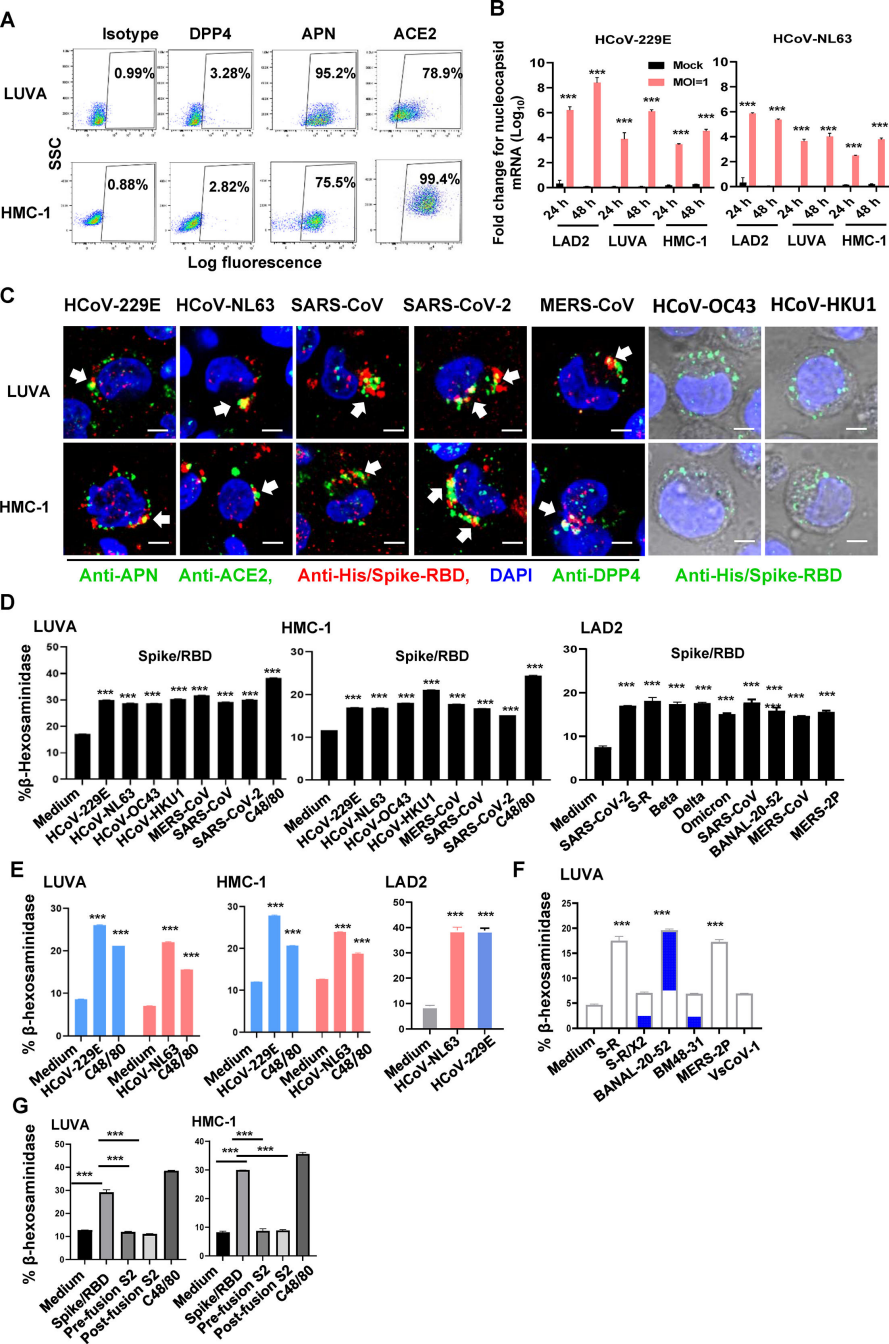
Binding of Spike/RBD proteins to receptors triggers MC degranulation. (**A**) Expression of ACE2, DPP4, and APN in LUVA and HMC-1 cells was detected by immunostaining with specific antibodies and analyzed with flow cytometry. (**B**) LUVA, LAD2, and HMC-1 cells (1 × 10^6^ cells for each) were treated with HCoV-229E and HCoV-NL63 (M.O.I = 1) or medium (mock) for 24 or 48 h. Viral replication was quantified by detecting the expression of nucleocapsid gene and normalizing with the gapdh gene. (**C**) HMC-1 and LUVA cells were exposed to Spike/RBD proteins (5 µg/mL) for 1 h at 4°C. The co-localization of these proteins with their respective receptors of APN, ACE2, and DPP4 was detected with confocal microscopy. Scale bar: 10 µm. (**D through G**) MC degranulation. MC degranulation in Spike/RBD protein-treated (**D**), HCoV-229E and HCoV-NL63 (M.O.I = 1) virus-infected LUVA, LAD2, or HMC-1 cells (**E**), Spike/RBD mutant protein-treated LUVA cells (**F**), and pre- and post-fusion S2 subunits from SARS-CoV-2-treated LUVA and HMC-1 cells (G) were detected by quantifying the β-hexosaminidase release. The compound 48/80 (C48/80) was used as the control. Data are presented as mean ± SD. One representative result from three independent repeats is shown. ***P < 0.001 is considered significant differences.

MCs express a diversity of membrane receptors that are capable of recognizing and responding to a multitude of stimuli (20). To elucidate the role of viral receptors in MCs, we first examined their expression profiles. We have recently found the expression of ACE2 on the LAD2 mast cell line, which facilitates the productive infection of SARS-CoV-2 (14). In this study, we extended the investigation to two additional human mast cell lines, namely LUVA and HMC-1. The analysis with flow cytometry exhibited that these cell lines expressed high levels of aminopeptidase N (APN) and ACE2, with a relatively lower expression of dipeptidyl peptidase 4 (DPP4) (Fig. 1A ;Fig. S1). Notably, the LAD2, LUVA, and HMC-1 mast cell lines were found to be susceptible to infection by human coronavirus 229E (HCoV-229E), which utilizes APN as its receptor, and human coronavirus NL63 (HCoV-NL63), which utilizes ACE2 as its receptor (Fig. 1B).

We have previously demonstrated that the binding of the Spike/RBD protein of SARS-CoV-2 to ACE2 receptor could trigger rapid MC degranulation (14). To further validate this observation and investigate the ubiquity for coronavirus-induced MC degranulation, we expanded the investigation to include recombinant Spike/RBD proteins sourced from multiple human coronaviruses. The binding of Spike/RBD proteins to cells was first investigated. Specifically, HMC-1 and LUVA cells were exposed to recombinant Spike/RBD proteins derived from HCoV-229E, HCoV-NL63, HCoV-OC43, HCoV-HKU1, Middle East respiratory syndrome coronavirus (MERS-CoV), SARS-CoV, and SARS-CoV-2 for 1 h at 4°C. The recombinant proteins were identified through immunostaining with a His-Tag antibody, and the receptors of APN, ACE2, and DPP4 were indicated with immunostaining with specific antibodies. Confocal microscopy was employed to observe the co-localization of these proteins with their respective receptors (Fig. 1C). The binding interactions of Spike/RBD proteins derived from human coronaviruses OC43 and HKU1, which recognize O-acetylated sialic acid (O-ac Sia) as their receptor, were visualized through the overlay of bright-field images with immunostaining of the His-tag (Fig. 1C).

MC degranulation was detected by measuring the secretion of granule content β-hexosaminidase (21). The compound 48/80 (C48/80) that can stimulate MC degranulation was used as the control. The treatment with human coronavirus Spike/RBD proteins induced degranulation in both HMC-1 and LUVA mast cell lines (Fig. 1D). We have previously demonstrated that LAD2 cells underwent rapid degranulation upon stimulation with Spike/RBD proteins or authentic viruses of SARS-CoV-2, HCoV-NL63, and HCoV-229E (14). Here, we extended this observation by examining the degranulation response of LAD2 cells to a broader range of Spike/RBD proteins derived from SARS-CoV-2 and its diverse mutants, including S-R, beta, delta, and omicron variants. Additionally, we included Spike proteins from other coronaviruses, including SARS-CoV, BANAL-20-52, MERS-CoV, and MERS-CoV-2P (Fig. 1D). Furthermore, we used the authentic viruses HCoV-NL63 and HCoV-229E to validate the triggered degranulation in HMC-1, LUVA, and LAD2 cells (Fig. 1E), reinforcing the finding that viral Spike proteins play a crucial role in initiating this process.

To ascertain whether the mere binding of Spike proteins to receptors on MCs is sufficient to trigger degranulation (14), these spike proteins derived from distinct coronaviruses that have the divergent binding affinity with their receptors were used. Specifically, modifications were introduced to the Spike protein of SARS-CoV-2, referred to as S-R, by covalently inserting a disulfide bond between residues 413 and 987. This engineered mutant, designated as S-R/x2, is stabilized in a prefusion conformation, which significantly attenuates its binding affinity for ACE2 (22, 23). The bat coronavirus BANAL-20-52 and the MERS-CoV-2P strain (specifically the England1 variant) showed high affinity binding to their respective dimeric receptors, ACE2 and DPP4 (24, 25). In contrast, the bat coronavirus BM48-31-CoV (GenBank ON131096) and VsCoV-1 exhibited limited binding ability to their receptors (26–28). Expectedly, the treatments with Spike/RBD proteins derived from S-R, BANAL-20-52, and MERS-CoV-2P, which maintain a high receptor binding affinity, resulted in the induction of MC degranulation (Fig. 1F). Conversely, Spike/RBD proteins isolated from S-R/x2, BM48-31, and VsCoV-1, which exhibited limited receptor binding capability, failed to elicit such an induction (Fig. 1F). Additionally, the S2 subunit from SARS-CoV-2 Spike protein was used as the control. These pre- and post-fusion S2 subunits do not contain the RBD domain and did not induce degranulation of LUVA and HMC-1 cells (Fig. 1G).

Collectively, these findings underscore the capacity and ubiquity of coronavirus Spike/RBD proteins to provoke MC degranulation and implicate that the interaction between Spike proteins and receptors is the pivotal trigger of this process.

### Transcriptome analysis reveals Spike/RBD protein-induced MC activation

To comprehensively elucidate the activation of MCs in response to stimuli with Spike/RBD proteins, we conducted a transcriptome analysis. LAD2 cells were exposed to Spike/RBD proteins derived from MERS-CoV, SARS-CoV, and SARS-CoV-2, as well as virions of HCoV-229E and HCoV-NL63, respectively, for 24 h. Transcriptome profiles of the LAD2 cells were analyzed using standardized protocols. Data from three independent repeats were summarized.

**Fig 2:**
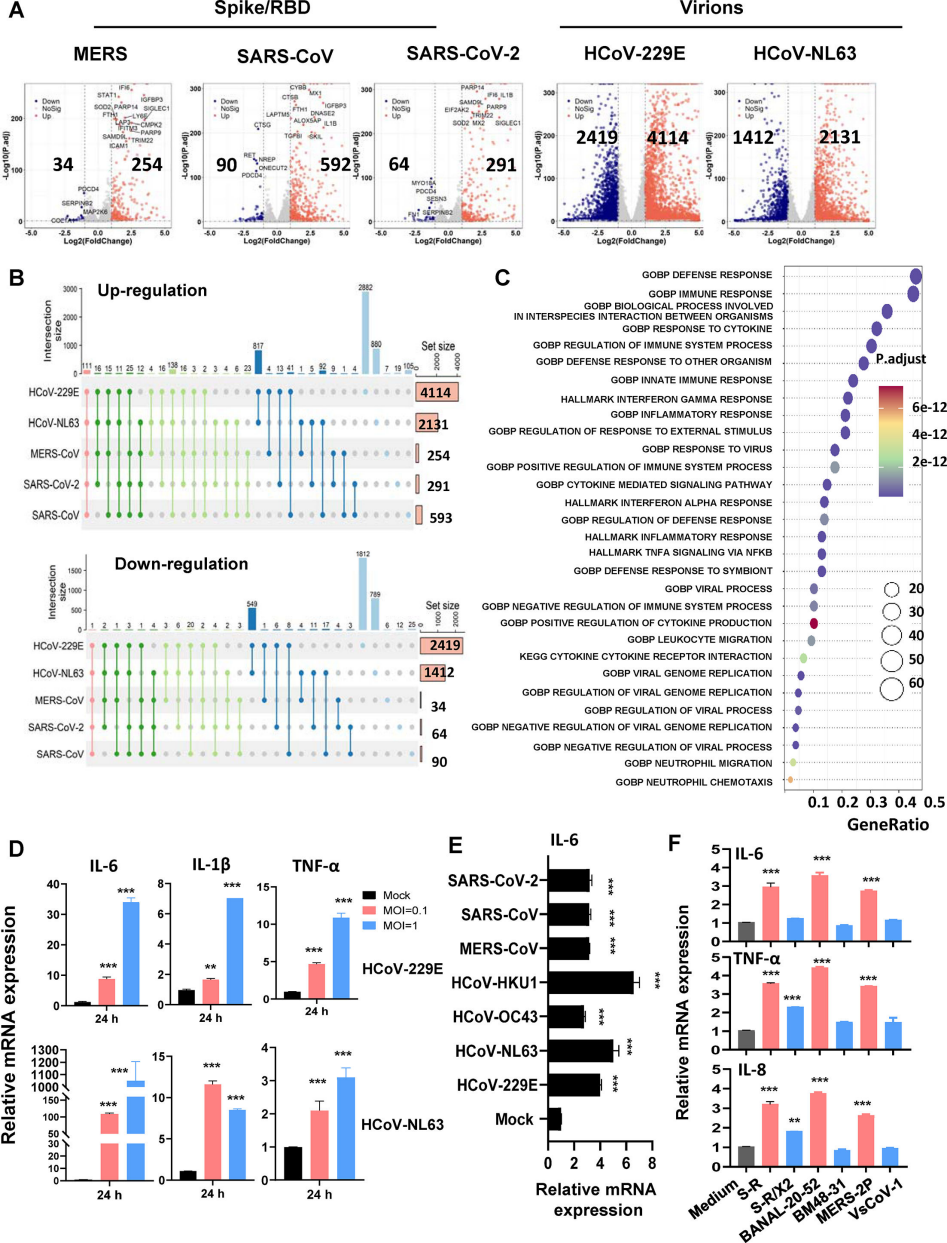
Transcriptome analysis reveals Spike/RBD proteins-induced MC activation. LAD2 cells were exposed to Spike/RBD proteins (5 µg/mL) or virions of HCoV-229E and HCoV-NL63 (MOI = 1) for 24 h. Total RNAs were extracted from cells, and the transcriptome analysis was conducted. Data from three independent repeats were summarized. (**A**) Volcano plot of DEGs comparing Spike/RBD proteins treated or HCoV-229E and HCoV-NL63 infected cells to that of mock-infection or medium-treatment. The symbols of top upregulated or downregulated genes are shown. (**B**) Summary of DEGs. The consistently upregulated and downregulated genes in LAD2 cells from these treatments with spike/RBD proteins and viral particles. (**C**) GO functional enrichment analysis of DEGs. The color bar indicates the minus logarithm of *Q* values, and bubble size indicates the absolute gene counts enriched in a GO term. (**D-F**) Expression of inflammatory factors. Total RNAs from LAD2 cells or LUVA cells were isolated. The expression of IL-1β, IL-6, TNF-α, or IL-8 in virus-infected LAD2 cells (**D**) or Spike/RBD protein-treated LUVA cells (**E, F**) were detected by real-time (RT-)PCR. Data are presented as mean ± SD. One representative result from three independent repeats is shown. *P < 0.05, **P < 0.01 and ***P < 0.001 are considered significant differences.

The analysis with the volcano plot revealed that treatment with Spike/RBD proteins led to the downregulation of a relatively limited number of genes, whereas it induced the upregulation of hundreds of genes (Fig. 2A). In contrast, the treatments with virions of HCoV-229E and HCoV-NL63 showed a profound effect on transcripts, causing thousands of differently expressed genes (DEGs) (Fig. 2A). A comprehensive analysis was conducted to summarize the genes that underwent consistent alterations in response to treatments involving Spike/RBD proteins or virions. A total of 111 genes were observed to be upregulated, whereas only a single gene, CLEC12A (C-type lectin domain family 12 member A), exhibited downregulation (Fig. 2B) (Fig. S2, Table S2).

To further elucidate the functional implications of these upregulated genes, a gene ontology (GO) functional enrichment analysis was performed. The genes that underwent consistent alterations in response to treatments involving Spike/RBD proteins (from MERS-CoV, SARS-CoV, and SARS-CoV-2) and virions (HCoV-229E and HCoV-NL63) were used for analysis. This analysis demonstrated an enrichment of gene sets that regulate immune response, cytokine production, and inflammatory responses among the upregulated genes (Fig. 2C;Fig. S2). The most prominently upregulated genes included CCL2, CCL3, CCL20, CCL3L1, CCL4, CXCL1, CXCL11, CXCL8, IL1β, IL18R1, IL32, IRAK2, IRF7, TNFAIP6, NFRSF9, and TNFSF15, etc. (Tables S2 and S3 ).

To validate the transcriptome data and quantify the upregulation of inflammatory factors, we performed real-time (RT-) PCR analysis. The results confirmed the upregulation of IL-1β, IL-6, and TNF-α in virus-infected LAD2 cells (Fig. 2D) or Spike/RBD protein-treated LUVA cells (Fig. 2E). Notably, when LUVA cells were treated with Spike/RBD proteins exhibiting limited receptor binding affinity, the induction of IL-6, TNF-α, and IL-8 levels was compromised (Fig. 2F), further underscoring the necessity of Spike protein binding to receptors for eliciting MC activation. In summary, these transcriptome data reveal Spike/RBD proteins-induced MC activation.

### Spike/RBD protein induces activation of the cellular Src/PI3K/AKT signaling pathway

In the subsequent analysis, we delved into the cellular pathways that are activated in response to Spike/RBD protein-induced MC degranulation. Despite the expressions of numerous membrane receptors that mediate MC activation upon stimulation, the most extensively studied mechanism underlying MC activation pertains to IgE-mediated FcεRI activation in type I hypersensitivity allergic reactions (1, 8). Upon binding of IgE to FcεRI, SFK member Lyn becomes engaged and undergoes phosphorylation for activation. This activation subsequently triggers a series of cytoplasmic cascades, ultimately culminating in the release and new synthesis of mediators within the cell (1, 8, 19).

**Fig 3:**
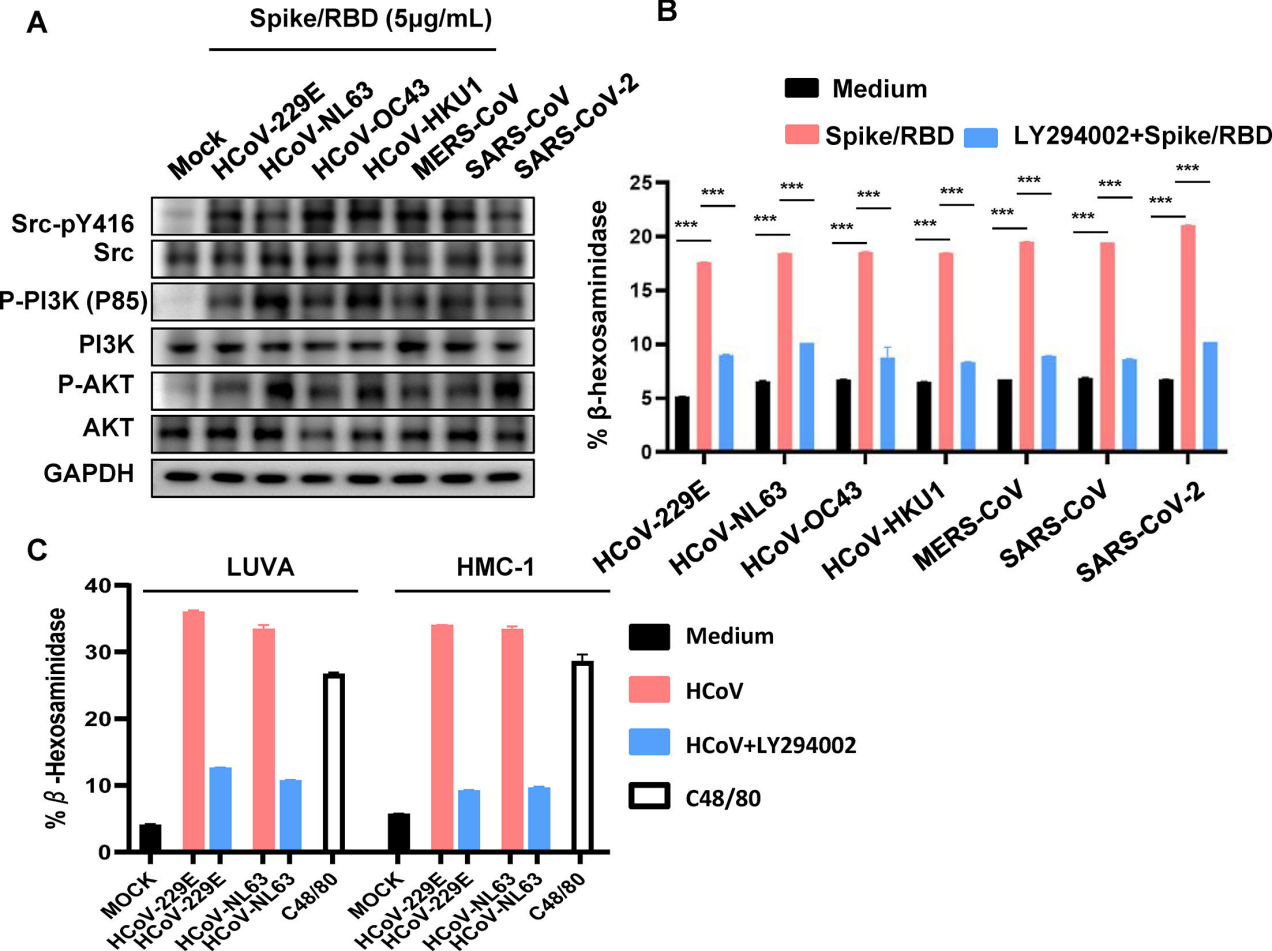
Spike/RBD proteins induce activation of the cellular Src/PI3K/AKT signaling pathway. (**A**) LUVA cells were exposed to Spike/RBD proteins (5 µg/mL) for 2 h. Src-pY416, Src, P-PI3K(P85), PI3K, P-AKT, and AKT levels were assessed by immunoblot analysis. (**B**) LUVA cells were exposed to Spike/RBD proteins (5 µg/mL) in the presence or not of LY294002 (100 µM) for 2 h. (**C**) LUVA or HMC-1 cells were treated with HCoV-229E and HCoV-NL63 (M.O.I = 1) for 2 h. MC degranulation was detected by quantifying the β-hexosaminidase release. Data are presented as mean ± SD. One representative result from three independent repeats is shown. ***P < 0.001 is considered significant differences.

Similarly, the treatment with Spike/RBD proteins was found to activate the cellular Src/PI3K/AKT signaling pathway. The treatments with Spike/RBD proteins elicited auto-phosphorylation of endogenous Src kinase at the tyrosine residue 416 (Tyr-416) (Fig. 3A). Further analysis of downstream cellular signaling cascades revealed an augmented phosphorylation of the p85 subunit of PI3K kinase, as well as Akt (Fig. 3A). Notably, when the PI3K inhibitor LY294002 was incorporated into the treatment alongside Spike/RBD proteins, the resultant degranulation of LUVA was completely abrogated (Fig. 3B). The LY294002 could also effectively block the degranulation of LUVA and HMC-1 cells induced by HCoV-229E and HCoV-NL63 virions (Fig. 3C). These findings underscore the necessity of the intracellular Src/PI3K/Akt signaling pathway for Spike protein-induced MC degranulation.

### Reducing the cytoplasmic Ca^2+^ or blocking the microtubule reorganization abolishes Spike protein-triggered MC degranulation

**Fig 4:**
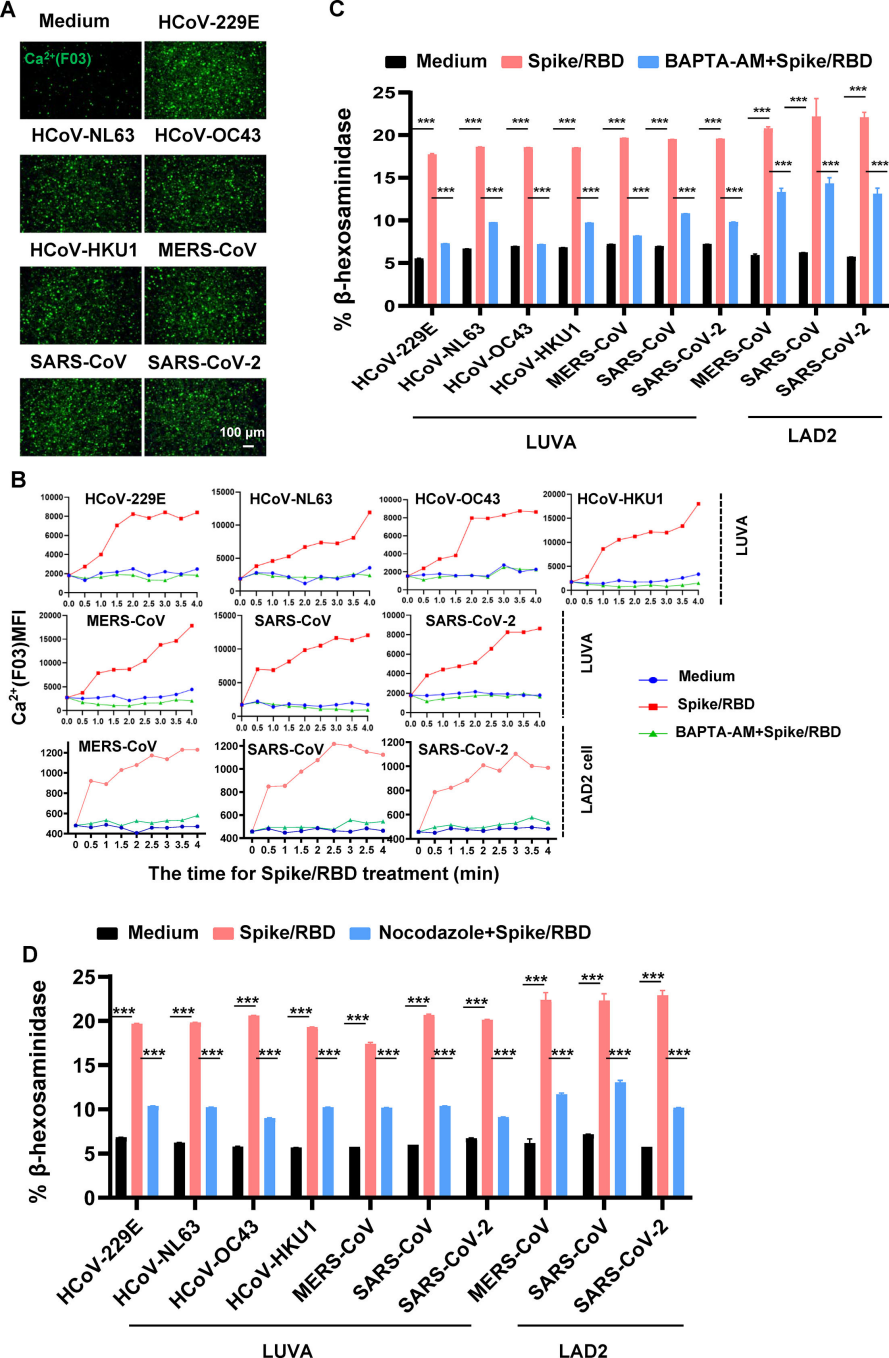
The role of cytoplasmic Ca^2+^ and microtubule reorganization in Spike protein-triggered MC degranulation. (**A**) LUVA cells were exposed to Spike/RBD proteins (5 µg/mL) for 2 h, and the F03 intracellular calcium ion fluorescent probe was added during the treatments. Cells were observed with confocal microscopy. Scale bar: 100 µm. (**B**) LUVA and LAD2 cells were exposed to Spike/RBD proteins (5 µg/mL) in the presence or not of BAPTA-AM (20 µM) for the indicated time, and the dynamic change of intracellular Ca^2+^ was monitored. (**C, D**) MC degranulation. LUVA and LAD2 cells were exposed to spike/RBD proteins (5 µg/mL) in the presence of BAPTA-AM (20 µM) (**C**) or Nocodazole (20 µM) (**D**) for 2 h. MC degranulation was detected by quantifying the β-hexosaminidase release. Data are presented as mean ± SD. One representative result from four independent repeats is shown. ***P < 0.001 is considered significant differences.

In the downstream signaling cascade triggered by FcεRI, both Ca²^+^-dependent and -independent pathways can mediate the reorganization of microtubules and actin filaments, which are essential for the transport of secretory granules and the subsequent membrane fusion (1, 8, 19). To investigate whether Spike protein-triggered MC degranulation is Ca^2+^-dependent, the F03 intracellular calcium ion fluorescent probe was added during the treatment of MCs with Spike/RBD proteins. An obvious increase in cytoplasmic Ca²^+^ concentration was observed after the treatments with spike/RBD proteins in LUVA cells (Fig. 4A). The rapid increases of cytoplasmic Ca^2+^ along with treatments were observed in both LUVA and LAD2 cells (Fig. 4B).

To investigate the Ca²^+^ dependency of this process, the Ca²^+^ chelator BAPTA-AM was used. The Ca²^+^ ion could be effectively chelated by BAPTA-AM (Fig. 4B). Subsequently, the induction of degranulation in both LUVA and LAD2 cells was abolished in the presence of BAPTA-AM (Fig. 4C), suggesting a crucial role of cytoplasmic Ca²^+^ in this process. The addition of nocodazole, a compound known to block microtubule reorganization, significantly diminished the triggered degranulation in both LUVA and LAD2 cells (Fig. 4D), demonstrating the dependence of microtubule reorganization for this process. Collectively, these data provide evidence that Spike protein-induced MC degranulation is dependent on both the elevation of cytoplasmic Ca²^+^ and the sequential reorganization of microtubules.

## DISCUSSION

**Fig 5:**
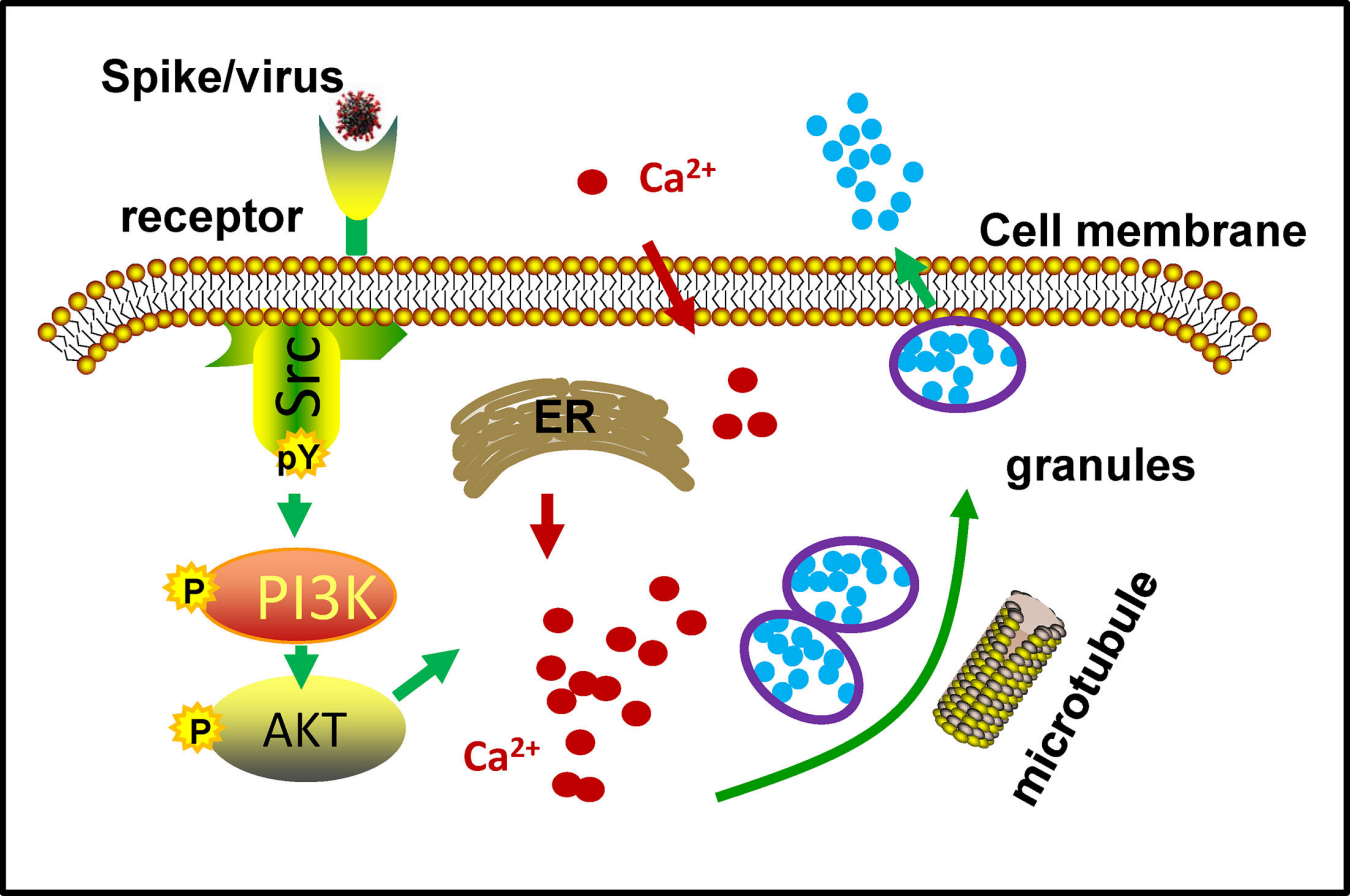
Graphical illustration of MC degranulation triggered by Spike/RBD proteins. The interaction between spike/RBD proteins and cellular receptors initiates the activation of src kinase, which subsequently stimulates the PI3K/AKT signaling pathway. This, in turn, leads to an accumulation of intracellular calcium ions. The elevated calcium levels facilitate microtubule-dependent granule transport, ultimately promoting MC degranulation.

In this study, we investigated the ubiquity of coronavirus-induced MC degranulation and the intracellular signaling cascades that mediate the activation of MCs. Our findings reveal that MCs can undergo degranulation upon stimulation with spike/RBD proteins derived from a variety of coronavirus strains. Mechanistically, the interaction between Spike/RBD proteins and cellular receptors initiates the activation of src kinase, which subsequently stimulates the PI3K/AKT signaling pathway. This, in turn, leads to an accumulation of intracellular calcium ions. The elevated calcium ions facilitate microtubule-dependent granule transport, ultimately promoting MC degranulation (Fig. 5). Our study sheds light on the mechanism of virus-triggered activation of MCs.

In this study, we have successfully detected the expression of receptors for DPP4, APN, and ACE2 in MCs. However, due to the unavailability of commercial antibodies, we were unable to detect the presence of the O-ac Sia receptor in MCs. Besides being the cellular receptors for viral fusion and sequential productive infection, these receptors, including the O-ac Sia, have been implicated as multifunctional modulators of immune cells, such as immune responses, inflammatory cytokine production, inflammatory cell adhesion, phagocytosis, autophagy, and cell death, etc (29–37). Src kinase-mediated tyrosine phosphorylation of APN regulates inflammatory cell–cell adhesion and monocyte trafficking (36). Our finding reveals the pivotal function of spike-receptor binding in initiating MC degranulation. This discovery offers fresh insights into the cellular functional modulation mediated by this Spike–receptor interaction.

We compared the mechanisms governing MC degranulation induced by viruses, particularly coronaviruses, and those triggered by the FcεRI receptor. In type I hypersensitivity allergic reactions, the interaction of IgE with FcεRI initiates receptor dimerization, leading to the engagement of SFK member Lyn. This engagement results in phosphorylation of FcεR subunits and sequential recruitment of ZAP-70 family Syk tyrosine kinase to initiate the phosphorylation of various intracellular targets, particularly the phospholipase Cγ (PLCγ). PLCγ subsequently catalyzes the hydrolysis of phosphatidylinositol 4,5-bisphosphate to produce the inositol 1,4,5-trisphosphate (IP3), which binds to the endoplasmic reticulum, thereby facilitating the efflux of Ca^2+^ ions (1, 8, 19). Finally, the increased cytoplasmic Ca^2+^ triggers cytoskeleton reorganization that is required for secretory granule transport and membrane fusion (19). Alternatively, the FcεRI signaling pathways also encompass a Ca^2+^-independent pathway (19). In this study, coronaviruses were found to elicit the activation of the src member of the SFKs. It is plausible that Lyn may also have been activated in response to the treatment with Spike proteins, albeit this requires further elucidation. Additionally, the degranulation of MCs triggered by the Spike protein is dependent on Ca^2+^ ions. There may be overlapping components within the cellular signaling cascades during these two kinds of stimuli but not being exactly the same.

Our transcriptome analysis provided a comprehensive view of the transcriptional alterations induced by Spike/RBD proteins. Future studies will focus on elucidating the functional significance of the upregulated genes and pathways. A notable observation was the consistent downregulation of the CLEC12A gene across various stimulations involving Spike/RBD proteins and viral particles. CLEC12A acts as an inhibitory receptor to play a crucial role in negatively regulating cellular activation to prevent inappropriate immune responses (38). Whether the downregulation of CLEC12A contributes to MC activation is worthy of further study.

In addition to coronaviruses, MCs can recognize and respond to a diversity of pathogens. These pathogens include but are not limited to bacteria, parasites, fungi, as well as various viruses, such as flaviviruses, respiratory viruses, retroviruses, hepatitis viruses, and even oncolytic viruses (6). It is noteworthy that, although pathogen-induced MC activation does not invariably result in degranulation, MC degranulation triggered by certain pathogens is capable of eliciting hyperinflammation and subsequent tissue damage, which may represent a prevalent mode of pathogenesis. For the evidence, the released chymase and tryptase from MCs triggered by Dengue virus infection break down the endothelial cell tight junctions and induce vascular permeability (39). Our previous research has found that histamine released from MCs enhances HIV-1-induced functional polarization of dendritic cells, resulting in immunosuppression through the stimulation of regulatory T-cell differentiation (40). Furthermore, MuLV/Friend virus-induced MC degranulation has been shown to activate granulocyte-like myeloid-derived suppressive cells in mice, which inhibit CD8^+^ T cell- and NK cell-mediated antiviral immune responses (21). Elucidating the dual roles of MCs in immune inflammation, both beneficial and detrimental, advances our understanding of viral pathogenesis and facilitates the development of effective antiviral therapeutic strategies.

In conclusion, our study provides insights into the mechanisms underlying virus-triggered activation of MCs. Elucidating the cellular mechanisms responsible for virus-induced MC activation and degranulation offers promising prospects for the development of MC-targeted antiviral therapeutic strategies.

## MATERIALS AND METHODS

### Cells, proteins, and viruses

HMC-1 cells were cultured in RPMI-1640 medium (Gibco) containing 10% bovine serum (Gibco) and supplemented with 100 U/mL penicillin and 100 µg/mL streptomycin (Invitrogen). LUVA cells were grown in StemPro-34 medium (Gibco) supplemented with 100 µg/mL nutrient supplement (Gibco), 100 U/mL penicillin, 100 µg/mL of streptomycin (Invitrogen), and 2 mM GlutaMAX (Gibco). LUVA and HMC-1 were purchased from Meisen CTCC, Zhejiang, China. HMC-1 cells are derived from the peripheral blood of a patient with mast cell leukemia (41), and LUVA cells are grown from CD34^+^-enriched mononuclear cells derived from the peripheral blood of a donor with aspirin-exacerbated respiratory disease (42). LAD2 (Huzhen Company, Shanghai, China) was cultured in StemPro-34 medium (Gibco) supplemented with 100 µg/mL stem cell factor (Novoprotein), 100 µg/mL interleukin-6 (IL-6) (Novoprotein), nutrient supplement (NS) (Gibco), 100 U/mL penicillin (Invitrogen), 100 µg/mL of streptomycin (Invitrogen), and 2 mM L-Glutamine (Gibco).

The recombinant HCoV-NL63 Spike/RBD (receptor-binding domains) (40600-V08H), MERS-CoV Spike/RBD protein (40071-V08B1), SARS-CoV-2 Spike/RBD (40592-V08B), HCoV-229E Spike/RBD (40601-V08H), HCoV-HKU1 Spike/RBD (40021-V08H), HCoV-OC43 (40607-V08H1), and SARS-CoV Spike/RBD (40150-V08B2) were purchased from Sino Biological, Beijing, China. The coronavirus HCoV-NL63 (NR-470) and HCoV-229E (VR-740) were purchased from the American Type Culture Collection.

### Confocal microscopy

Cells were seeded on the coverslips (CORNING, 354085) and treated with the recombinant coronavirus Spike/RBDs (5 µg/mL) at 4°C for 1 h. Cells were then fixed with 4% paraformaldehyde (Biosharp, BL539A) for 30 min, permeabilized in 0.1% Triton X-100 (Solarbio, T8200), and incubated with the primary antibody (Anti-His M30111L, Abmart; Anti-ACE2 21115–1-AP, Proteintech; Anti-APN PA5-109314, Invitrogen; Anti-DPP4 AF1180, R&D; Anti-His PA5-117499, Invitrogen) for 1 h at room temperature. Cells were washed three times with phosphate-buffered saline (PBS), then labeled with the appropriate fluorescent secondary antibodies (Alexa Fluor 488-conjugated goat anti-rabbit IgG, A-11034, Alexa Fluor 568-conjugated goat anti-mouse IgG, A-11004, Thermo Fisher Scientific, or Alexa Fluor 488 goat anti-mouse IgG, SA11001s, Invitrogen) for 1 h. The cells were then washed in PBS, labeled with DAPI (Invitrogen, P36971), and mounted with ProLong Diamond. Images were captured on a Zeiss LSM 800 confocal microscope and processed in ImageJ software.

### Mast cell degranulation

Mast cell lines (3 × 10^5^) were exposed to the recombinant coronavirus Spike/RBDs (5 µg/mL) or HCoV-NL63 (ATCC, NR-470) and HCoV-229E (ATCC, VR-740) (M.O.I = 1) for the indicated time. The compound 48/80 (C48/80) (4 µg/mL) (Sigma, C2313) was used as the control. In some samples, the nocodazole (20 µM) (HY-13520, MedChemExpress), BAPTA-AM (20 µM) (HY-100545, MedChemExpress), or LY294002 (100 µM) (HY-10108, MedChemExpress) were added. The cell culture supernatants were harvested, and the release of β-hexosaminidase was detected (21). The proportion of degranulation was determined through the division of the absorbance measured in the supernatant by the aggregate absorbance observed in both the supernatant and the cellular lysate.

### Detection of intracellular calcium

Cells (a cell density of 1 × 10^6^ /mL) were loaded with the F03 intracellular calcium ion fluorescent probe (Biorab, HR0943) at a ratio of 1:1,000. After gentle mixing, the cells were incubated at 37°C for 1 h. Then, the cells were treated with spike/RBDs or other stimulus for the indicated time. Images were taken using a fluorescent microscope and the appropriate bandpass FITC filter. An excitation wavelength of 488 nm was provided by a Zeiss LSM 800 confocal microscope, and fluorescence signals were examined using a 515 nM pass emission filter. Background fluorescence was subtracted from all signals. The average fluorescence intensity was measured by Image J software.

### Real-time (RT-) PCR

Total cellular mRNA from cells was extracted with Trizol reagent (Life Technologies) and was reversely transcribed to cDNA with ReverTra Ace qPCR RT Master Mix with gDNA Remover Kit (TOYOBO) according to the manufacturer’s protocol. Primers used in RT-PCR are listed in Table S1.

### Western blotting

Cells were lysed for 1 h at 4°C in lysis buffer (Beyotime) containing protease inhibitors and phosphorylation inhibitors. After centrifugation for 10 min at 12,000×*g*, the supernatant was boiled in reducing SDS sample loading buffer and subjected to 10% SDS-PAGE electrophoresis gel. After membrane transfer, these specific antibodies were used for immunoblotting: anti-phosphory PI3K/p85 (Cell Signaling Technology, 4228S), anti-PI3K/p85 (Cell Signaling Technology, 4257S), anti-phosphory AKT(T308) (Cell Signaling Technology, 13038S), anti-AKT (Cell Signaling Technology, 4619S), anti-Src Polyclonal (Proteintech, 11097–1-AP), anti-phospho-Src family (Tyr416) (Cell Signaling Technology, 6943), and anti-GAPDH (clone 3B3) (Abmart, M20006). The horseradish peroxidase (HRP)-conjugated goat anti-mouse IgG or goat anti-rabbit IgG (Sigma) as the secondary antibodies, and ECL luminescence reagent (Pierce) was used for visualization.

### RNA sequencing

LAD2 cells were exposed to Spike/RBD proteins or virions for 24 h, samples were collected, and total RNAs were extracted using Trizol (Invitrogen) and ribosomal RNA removed using QIAseq FastSelect-rRNA HMR Kits (QIAGEN). Fragmented RNAs (average length ~200 bp) were subjected to first-strand and second-strand cDNA synthesis, followed by adaptor ligation and enrichment with a low-cycle according to the instructions of NEBNext UltraTM RNA Library Prep Kit for Illumina (NEB, USA). The purified library products were evaluated using the Agilent 2200 TapeStation and Qubit2.0 (Life Technologies). The libraries were paired-end sequenced (PE150, Sequencing reads were 150 bp) at Guangzhou RiboBio Co., Ltd., using the Illumina HiSeq 3000 platform. Data analysis was performed according to references 14 and 17.

### Statistical analysis

GraphPad Prism 8.0 was used for statistical analysis. The statistical significance of the difference was determined through Student’s unpaired *t*-test.

## Data Availability

The raw sequence data reported in this paper have been deposited in the Genome Sequence Archive in National Genomics Data Center, China National Center for Bioinformation / Beijing Institute of Genomics, Chinese Academy of Sciences (GSA-Human: HRA007769), and are publicly accessible at https://ngdc.cncb.ac.cn/gsa.

## SUPPLEMENTAL MATERIAL

Figure S1 jvi.00078-25-s0001.tiff Expression of receptors.

Figure S2 jvi.00078-25-s0002.tiff GO functional enrichment analysis of DEGs.

Supplemental legends jvi.00078-25-s0003.docx Legends for supplemental material.

Table S1 jvi.00078-25-s0004.xlsx Primers.

Table S2 jvi.00078-25-s0005.xlsx Consistent up-regulated and downregulated genes.

Table S3 jvi.00078-25-s0006.xlsx Pathways of the 111 common upregulated genes.

ASM does not own the copyrights to Supplemental Material that may be linked to, or accessed through, an article. The authors have granted ASM a non-exclusive, world-wide license to publish the Supplemental Material files. Please contact the corresponding author directly for reuse.
