# Supplementary figures and images for: Spike proteins of coronaviruses activate mast cells for degranulation via stimulating Src/PI3K/AKT/Ca2+ intracellular signaling cascade
Source: J Virol. 2025 Apr 30;99(5):e00078-25. doi: 10.1128/jvi.00078-25 (PMC12090780; doi:10.1128/jvi.00078-25)

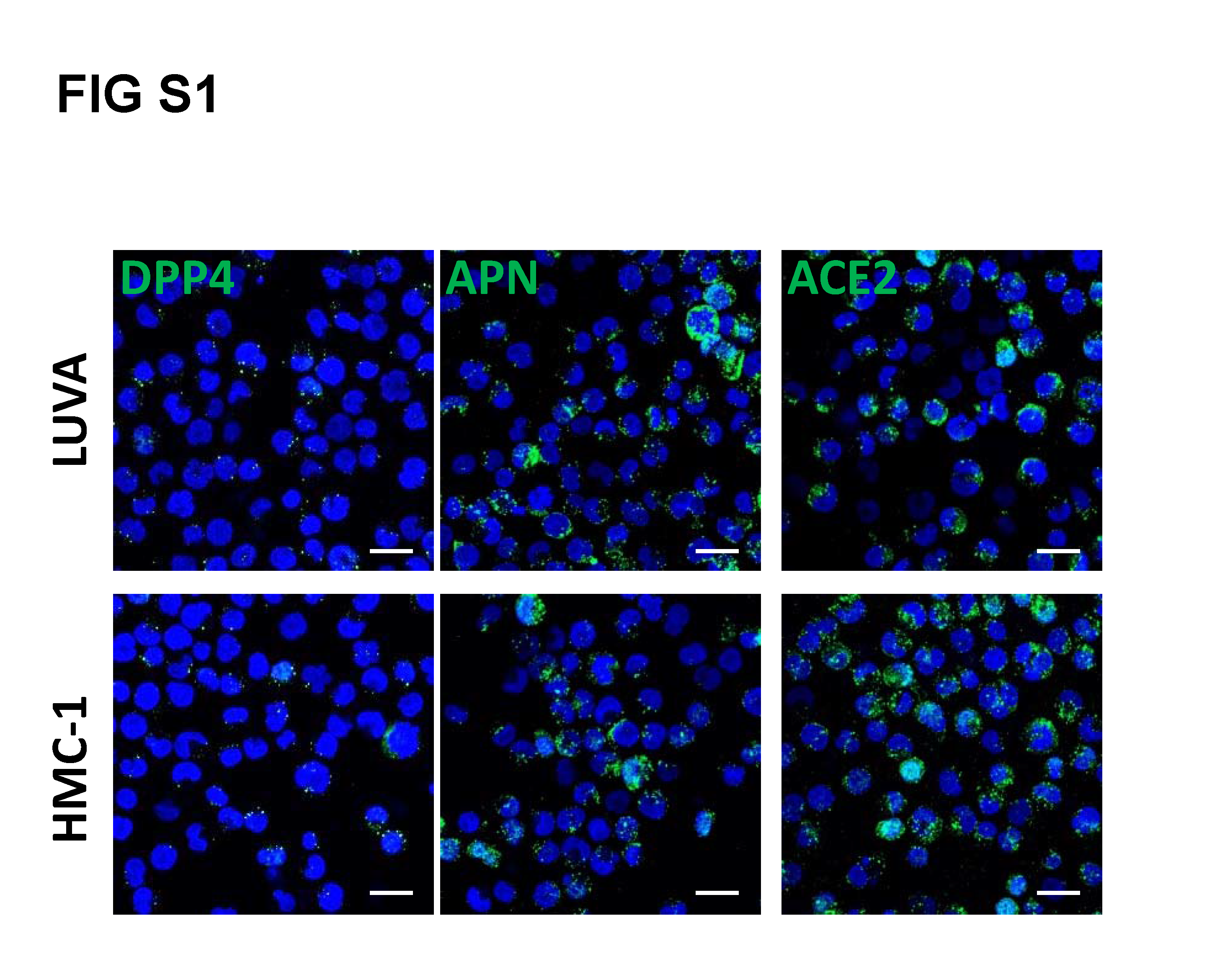

Supplement: Figure S1 — Expression of receptors. [file jvi.00078-25-s0001.tiff]

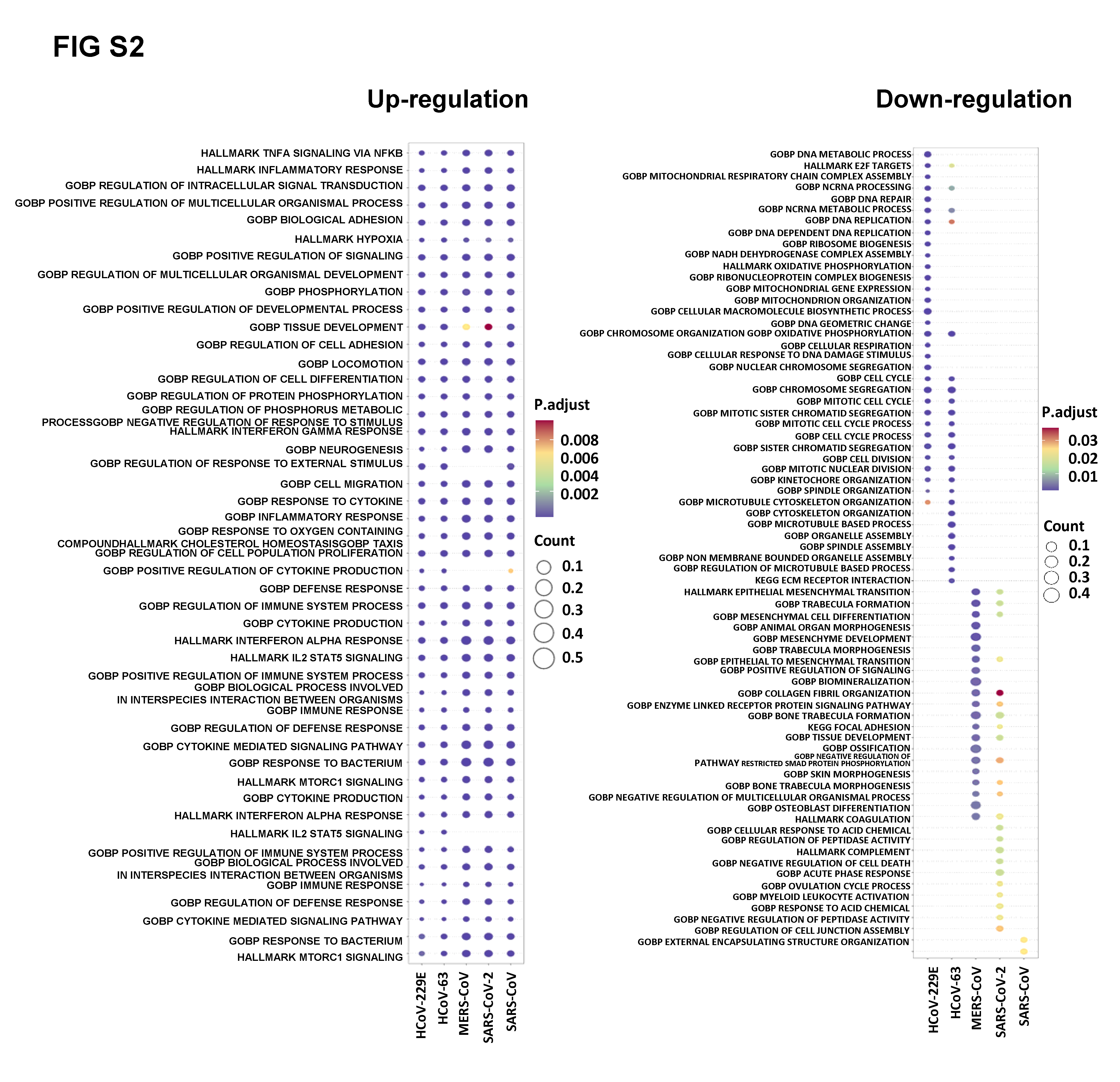

Supplement: Figure S2 — GO functional enrichment analysis of DEGs. [file jvi.00078-25-s0002.tiff]
