# Supplementary material for: Spike proteins of coronaviruses activate mast cells for degranulation via stimulating Src/PI3K/AKT/Ca2+ intracellular signaling cascade
Source: J Virol. 2025 Apr 30;99(5):e00078-25. doi: 10.1128/jvi.00078-25 (PMC12090780; doi:10.1128/jvi.00078-25)
Supplement: Supplemental legends — Legends for supplemental material. [file jvi.00078-25-s0003.docx]

**FIGURE LEDENDS**

**FIG S1. The expression of receptors.** The LUVA and HMC-1 cells were seeded on the coverslip, and the expression of DPP4, APN and ACE2 were detected with immunostaining with antibodies (indicated in green color). The cells were labeled with DAPI (indicated in blue color). Images were captured on a Zeiss LSM 800 confocal microscope and processed in ImageJ software. Scale bar: 100 µm.

**FIG S2. GO functional enrichment analysis of DEGs**. LAD2 cells were exposed to Spike/RBD proteins (5 μg/mL) or virions of HCoV-229E and HCoV-NL63 (M.O. I = 1) for 24 h. Total RNAs were extracted from cells and the transcriptome analysis were conducted. Data from 3 independent repeats were summarized. The DEGs in each treatment by either of Spike/RBD proteins or viral particles compared to the control of mock-infection or medium-treatment were conducted a GO analysis. The color bar indicates the minus logarithm of *q* values, and bubble size indicates the absolute gene counts enriched in a GO term.

**Suppl Excel 1.** Primers used in real-time (RT-) PCR.

**Suppl Excel 2.** The consistent up-regulated and down-regulated genes in transcriptome in LAD2 cells upon various stimulations.

**Suppl Excel 3.** Pathways for upregulated genes in transcriptome in LAD2 cells upon various stimulations.
